# Kuo-Hua Sun: The founder of physiologic psychology and child psychology in China

**DOI:** 10.1007/s13238-019-00666-w

**Published:** 2019-11-01

**Authors:** Yimeng Wang, Yanyan Qian

**Affiliations:** 1 School of Psychology, Nanjing Normal University, Nanjing, 210097 China; 2 Social and Behavioral Sciences Facility, Leiden University, 2333AK Leiden, The Netherlands

Kuo-Hua Sun (孙国华, 1902–1958, courtesy name—Xiao Meng) (Fig. 1) was a Chinese psychologist mainly engaged in Physiological Psychology and Child Psychology.

**Figure 1:**
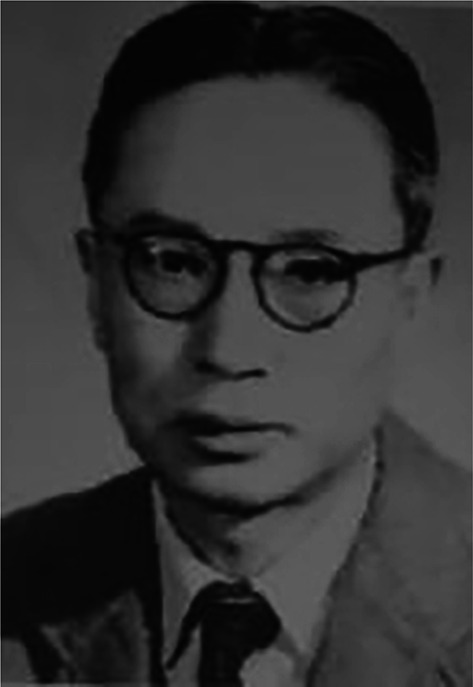
Kuo-Hua Sun

Kuo-Hua Sun was born on March 6th, 1902, in Weixian, Shandong province. He studied in Tsinghua School from 1914 to 1923. At the same year, Sun attended the education school of Ohio State University, and then received a Bachelor of Psychology (1928) and a Doctor of Philosophy (1929) from Ohio State University. During this period, Sun studied in the Department of Physiology at Chicago University (1925–1926) (Fig. 2). At the end of 1928, Sun returned to China. He was appointed as the dean and professor of the Psychology Department at Tsinghua University, and a professor of Beijing Normal University, Northeastern University, and Peking University. From 1941 to 1946 he temporarily left the teaching profession because of his illness; during this time he served as an editor and general affairs director in the former National Compilation Library. In 1952, Sun served as the deputy head of the Department of Philosophy and was a member of the school council of Peking University. He was also an academic member of the Institute of Psychology of the Chinese Academy of Sciences (CAS), a leader of the Department of Occurrence and Development Psychology, a director of the Chinese Psychological Association, and a managing editor of the *Journal of Psychology*. On February 8th, 1958, Sun died of heart attack at 56 years of age^1^ (Editorial Board of Acta Psychologica Sinica, 2003).

**Figure 2:**
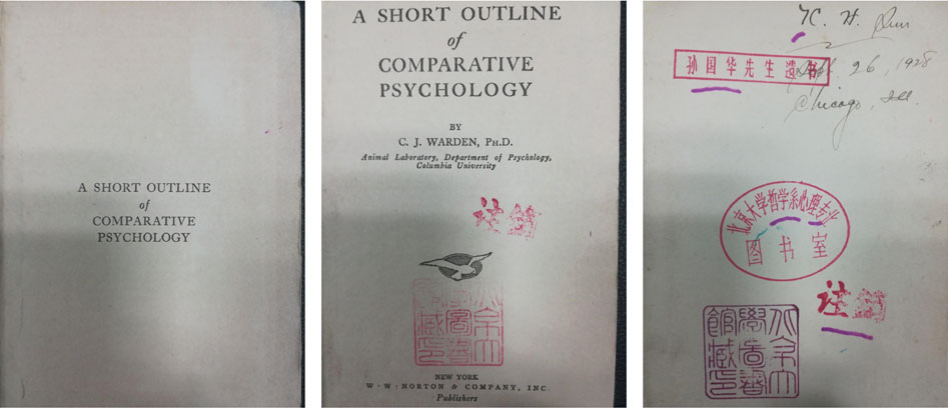
Sun bought the book when he studied in Chicago, USA

Sun had two main research interests throughout his life: Physiological Psychology and Child Psychology. In the field of Physiological Psychology, he published the paper “*The pupillary reflex in birds*” in *American Journal of Physiology* in 1926. He later published “*Do earthworms grow by adding segments*?” in *American Naturalist* (Sun and Pratt, 1931), and it has indeed commonly been asserted that one of the factors contributing to the growth in length of earthworms is the laying down of new segments. This paper, however, found that the number of links between two newly emerged worms (probably *Helodrillus foetidus*) was essentially the same as that among mature specimens of the species (Sun and Pratt, 1931). Three stages of development were selected for investigation: (1) Worms just emerging from cocoons; (2) Worms having no clitella (collected from the compost heap); (3) Worms selected on the basis of clitella-these being considered indicative of sexual maturity.” The results showed that: (1) As for the relationship between body length and segment number, there were significant differences among different stages of development, so whether earthworms can grow by increasing segments can be solved only when larvae with a certain number of segments can mature and recount; (2) Earthworms with zones (genital belts) do not belong to the same type as those without links (Sun and Pratt, 1931). Furthermore, according to Web of Science, this article was cited 13 times, with the most recent citation recorded in 2010.

In 1936, as an editor and publisher, Sun cooperated with Siegen K. Chou (周先庚) and Chih Wei Luh (陆志伟) founded the first bilingual journal of psychology—*The Chinese Journal of Psychology* in Beijing (Xin, 1991). Since then, Sun published dozens of articles in this journal, in cooperation with other researchers. Taking planaria research for example, Sun and Fwu Tarng Dun (敦福堂) (1936) wrote the report “*Note on Geotropism in the dark-adapted planaria gonocephala*”, arguing that *Planaria maculata* did not show a negative response to geocentric attraction when it found food. Additionally, the paper showed that the ability to resist confusion at the reversal of brightness values of the figure and the ground is used as a test for the perception of form. Sun and colleagues later investigated this in rats with the paper “*Brightness reversal in form discrimination of the rat as compared with a young child*” (Luh and Sun, 1936). In 1936, Sun and Chang Min-Chueh (张民觉) co-authored “*Feeding behavior of headless planaria dorotocephala*”. The study showed that *Planaria dorotocephala* still had a food response (Sun and Chang, 1936).

In the field of Child Psychology, Sun was conferred the master’s degree with his thesis “*Phylogenesis of the Sensorimotor System*” in the winter of 1927 (List of Graduate Degrees and Dissertations in Education Educational Research Bulletin, 1928). In 1928, Sun was awarded his doctor’s degree with his dissertation “*A study of visual and auditory reactions in infants*” (Sun, 1928).

In 1930, co-authoring with Karl Chapman Pratt and Amalie Kraushaar Nelson, Sun published “*The behavior of the newborn infant*” (Fig. 3) which explained the general developmental rules of the newborn infants’ behavior: “(1) The infants behavior was generalized, and any stimulation of the body in any place would lead to almost all parts of the individuals’ response; (2) The response of the stimulated parts was stronger than that of other parts, and the intensity and frequency of stimulation gradually decreased from the stimulated parts to the surrounding parts”. This article, printed in a single book for* Ohio State University Studies*: *Contributions in Psychology*, *Volume 10* (Pratt et al., 1930), was regarded as having great value in Child Psychology. To date, the Google Scholar search engine reports that the article has 162 citations. In 1933, the *Review of Educational Research* highly praised Sun, Pratt, and Nelson’s studies of psychological development of infants from birth to adolescence (Frank and George, 1933).

**Figure 3:**
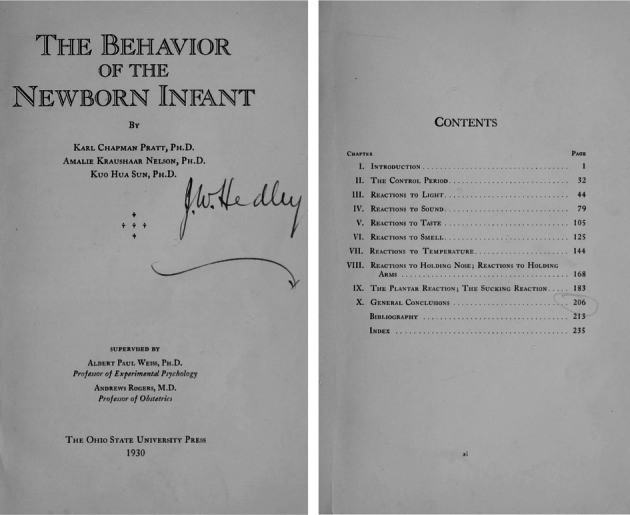
Dr. Sun composed the Chapter III, IV, V and VI of *The behavior of the newborn of infant*, and made efforts to descriptions and drawings of all the apparatus, preparation of the data, and coordination of the different sections of the monograph

Sun was also involved in foreign academic resource translation, including *The History of Psychology* (Pillsbury, 1929), *The Modern Cat*: *Her Mind & and Manners*: *An Introduction to Comparative Psychology* (Gates, 1928), *The Process of Human Behavior* (Sherman and Sherman, 1929), *An Introduction to Animal Psychology*: *The Behavior of the Rat* (Munn, 1933), and *The Sense and Will of Babies* (*Die Seele des Kindes*) (Preyer, 1889).

The construction of disciplines is another area of Sun’s significant work. In the autumn of 1926, in collaboration with other academics, Sun founded the psychology department of Tsinghua University, where Sun gave lectures regularly, alongside Yueh Tang (唐钺), Siengen K. Chou, Lv Shen (沈履), and Li Chen (陈立) (Qian and Li, 2011, p. 286). It was one of the earliest psychology departments in Chinese universities and one of the six major departments in the School of Science of Tsinghua University. In 1952, led by Sun, the deputy director of the philosophy department at Peking University, Jiao Shao (邵郊) joined this institute as Sun’s coadjutant and assisted Sun to adjust the division of disciplines, offer civil service, give lectures and conduct experiments on higher nervous system activity, animal psychology, and comparative neuroanatomy (Qian and Li, 2011; Wang, 2019). One year later, Sun, cooperating with physiological psychologists and comparative psychologists Jiao Shao (Wang et al., 2019) and Shen Nai Chang (沈迺璋), established the first Animal Conditioned Reflex Laboratory in China which later developed into the Physiological and Psychological Laboratory, which played a key role in psychology for becoming a national key discipline (Qian and Li, 2011; Wang et al., 2019). In the autumn of 1955, Sun founded the Child Psychology Laboratory in Peking University, which grown into the Developmental Psychology Laboratory of the Psychology Department in Peking University. This laboratory prompted plentiful researches in a leading position, especially in the area of children’s language.

## Footnotes

1 One recording of Kuo-hua Sun’s lifetime, another statement is 1902.01.26–1958.07.08 in Encyclopedia of China, Psychology Volume. (中国大百科全书(心理学卷) ).
